# Personality, Social Factors, Brain Functioning, Familial Risk, and Trajectories of Alcohol Misuse in Adolescence

**DOI:** 10.1001/jamanetworkopen.2024.25114

**Published:** 2024-08-16

**Authors:** Mira Tschorn, Laura Daedelow, Laura Szalek, Tobias Banaschewski, Arun L. W. Bokde, Sylvane Desrivières, Herta Flor, Antoine Grigis, Hugh Garavan, Penny Gowland, Jean-Luc Martinot, Marie-Laure Paillère Martinot, Eric Artiges, Frauke Nees, Dimitri Papadopoulos Orfanos, Luise Poustka, Sarah Hohmann, Christian Buechl, Michael N. Smolka, Nilakshi Vaidya, Henrik Walter, Robert Whelan, Gunter Schumann, Andreas Heinz, Michael A. Rapp

**Affiliations:** 1 Division of Social and Preventive Medicine, Department of Sports and Health Sciences, Intrafaculty unit of Cognitive Sciences, Faculty of Human Science, and Faculty of Health Sciences Brandenburg, Research Area Services Research and e-Health, University of Potsdam, Potsdam, Germany; 2 German Center for Mental Health (DZPG), Berlin and Potsdam, Germany; 3 Department of Psychiatry and Psychotherapy Campus Charité Mitte, Charité–Universitätsmedizin Berlin, corporate member of Freie Universität Berlin, Humboldt-Universität zu Berlin, Berlin Institute of Health, Berlin, Germany; 4 Department of Psychology, Humboldt-University Berlin, Germany; 5 Department of Child and Adolescent Psychiatry and Psychotherapy, Central Institute of Mental Health, Medical Faculty Mannheim, Heidelberg University, Mannheim, Germany; 6 Discipline of Psychiatry, School of Medicine, Trinity College Institute of Neuroscience, Trinity College, Dublin, Ireland; 7 Centre for Population Neuroscience and Precision Medicine, Institute of Psychiatry, Psychology & Neuroscience, Social, Genetic, and Developmental Psychiatry Centre, King’s College London, London, United Kingdom; 8 Institute of Cognitive and Clinical Neuroscience, Central Institute of Mental Health, Medical Faculty Mannheim, Heidelberg University, Mannheim, Germany; 9 Department of Psychology, School of Social Sciences, University of Mannheim, Mannheim, Germany; 10 NeuroSpin, CEA, Université Paris-Saclay, Gif-sur-Yvette, France; 11 Department of Psychiatry, University of Vermont, Burlington; 12 Department of Psychology, University of Vermont, Burlington; 13 Sir Peter Mansfield Imaging Centre School of Physics and Astronomy, University of Nottingham, University Park, Nottingham, United Kingdom; 14 Institut National de la Santé et de la Recherche Médicale, INSERM U 1299 Trajectoires Développementales & Psychiatrie, Ecole Normale Supérieure Paris-Saclay, Centre Borelli, University Paris Saclay, Centre National de la Recherche Scientifique, Gif-sur-Yvette, France; 15 Department of Child and Adolescent Psychiatry, Pitié-Salpêtrière Hospital, Assistance Publique–Hôpitaux de Paris Sorbonne University, Paris, France; 16 Institut National de la Santé et de la Recherche Médicale, Ecole Normale Supérieure Paris-Saclay, University Paris Saclay, Centre Borelli, CNRS, and Psychiatry Department, Établissement Public de Santé Barthélémy Durand, Etampes, France; 17 Institute of Medical Psychology and Medical Sociology, University Medical Center Schleswig Holstein, Kiel University, Kiel, Germany; 18 Department of Child and Adolescent Psychiatry and Psychotherapy, University Medical Centre Göttingen, Göttingen, Germany; 19 Department of Psychiatry and Psychotherapy, Technische Universität Dresden, Dresden, Germany; 20 Centre for Population Neuroscience and Stratified Medicine (PONS), Department of Psychiatry and Neuroscience, Charité Universitätsmedizin Berlin, Germany; 21 School of Psychology, Global Brain Health Institute, Trinity College Dublin, Dublin, Ireland; 22 Centre for Population Neuroscience and Precision Medicine (PONS), Institute for Science and Technology of Brain-Inspired Intelligence (ISTBI), Fudan University, Shanghai, China

## Abstract

**Importance:**

The development of an alcohol use disorder in adolescence is associated with increased risk of future alcohol dependence. The differential associations of risk factors with alcohol use over the course of 8 years are important for preventive measures.

**Objective:**

To determine the differential associations of risk-taking aspects of personality, social factors, brain functioning, and familial risk with hazardous alcohol use in adolescents over the course of 8 years.

**Design, Setting, and Participants:**

The IMAGEN multicenter longitudinal cohort study included adolescents recruited from European schools in Germany, the UK, France, and Ireland from January 2008 to January 2019. Eligible participants included those with available neuropsychological, self-report, imaging, and genetic data at baseline. Adolescents who were ineligible for magnetic resonance imaging or had serious medical conditions were excluded. Data analysis was conducted from July 2021 to September 2022.

**Exposure:**

Personality testing, psychosocial factors, brain functioning, and familial risk of alcohol misuse.

**Main Outcome and Measures:**

Hazardous alcohol use as measured with the Alcohol Use Disorders Identification Test scores, a main planned outcome of the IMAGEN study. Alcohol misuse trajectories at ages 14, 16, 19, and 22 years were modeled using latent growth curve models.

**Results:**

A total of 2240 adolescents (1110 female [49.6%] and 1130 male [50.4%]) were included in the study. There was a significant negative association of psychosocial resources (β = −0.29; SE = 0.03; *P* < .001) with the general risk of alcohol misuse as well as a significant positive association of the risk-taking aspects of personality with the intercept (β = 0.19; SE = 0.04; *P* < .001). Furthermore, there were significant positive associations of the social domain (β = 0.13; SE = 0.02; *P* < .001) and the personality domain (β = 0.07; SE = 0.02; *P* < .001) with trajectories of alcohol misuse development over time (slope). Family history of substance misuse was negatively associated with general risk of alcohol misuse (β = −0.04; SE = 0.02; *P* = .045) and its development over time (β = −0.03; SE = 0.01; *P* = .01). Brain functioning showed no significant association with intercept or slope of alcohol misuse in the model.

**Conclusions and Relevance:**

The findings of this cohort study suggest known risk factors of adolescent drinking may contribute differentially to future alcohol misuse. This approach may inform more individualized preventive interventions.

## Key Points

**Question:**

Are there differential associations of personality, social factors, brain functioning, and familial risk with alcohol misuse (AM) trajectories in adolescents?

**Findings:**

In this cohort study of 2040 adolescents that investigated alcohol misuse trajectories over the course of 8 years, psychosocial resources (lower frequency of life events realted to family and sexuality and higher socioeconomic status) were initially associated with a lower general risk for alcohol misuse but with an increased risk over the course of 8 years. Personality characteristics (higher impulsivity, risk-taking, and extraversion) were associated with a higher risk of alcohol misuse on average, as well as a higher risk for alcohol misuse development; there was no association with brain functioning.

**Meaning:**

These findings indicate that known risk factors contribute differentially to future AM, which may inform individualized interventions.

## Teaser

This cohort study uses latent growth curve modeling to analyze associations of personality, social factors, brain functioning, and family history with alcohol misuse over time among adolescents in Europe.

## Introduction

The worldwide lifetime prevalence of alcohol use is 80% and 10.7% for alcohol use disorder (AUD),^1^ suggesting that only a portion of users develop AUD. In young adolescents, rates of weekly alcohol use vary between 9% and 16%,^2^ and alcohol use in adolescence may result in physical injury, high-risk sexual behavior, long-term adverse neurocognitive effects, and an enhanced risk of developing AUD in adult life.^3^ Therefore, early identification of risk factors is crucial to develop interventions to prevent AUD. The most relevant risk factors are reward-related brain functioning, genetics, personality, and social factors.^4^ Personality and social environment factors, including parental drinking and parental provision of alcohol, are associated with alcohol misuse in early adolescence and in the development of adolescent alcohol use.^5,6^ Peer and parental influences on alcohol misuse seem to decrease in adulthood, whereas environmental factors seem to change over time.^7^ In a recent study examining markers of binge drinking,^8^ the neuropsychosocial and the psychosocial model outperformed the neural model for predicting risky drinking behaviors. While functional and structural brain measures are associated with alcohol use,^9^ these factors demonstrated modest utility, and the development of a neuroimaging risk score to yield targeted interventions is ongoing.^9^ Still, a comprehensive review on the imaging genetics results of the IMAGEN study by Mascarell Maričić et al^10^ found various effects of genetics on intermediate phenotypes. Therefore, the aim of the current study is to quantify the risk for alcohol misuse and subsequent dependency with respect to such intermediate phenotypes and associated domains for the first time in a longitudinal design with participants aged 14 to 22 years in the IMAGEN sample.

## Methods

In this cohort study, we used baseline data from participants from the longitudinal IMAGEN cohort^11^ that started with 14-year-old students from 8 sites in Germany, the UK, France, and Ireland. All local ethics research committees approved the study in accordance with the Declaration of Helsinki.^12^ This study followed the Strengthening the Reporting of Observational Studies in Epidemiology (STROBE) reporting guideline. Verbal or written consent was obtained from the parent or guardian and the participants depending on their age. Participants were recruited in schools with a maximization of ethnic homogeneity of European descent and sample diversity regarding sociodemographic variables and behavioral and emotional functioning. Exclusion criteria were ineligibility for magnetic resonance imaging scans and serious medical conditions. Alcohol misuse trajectories were assessed at 4 consecutive time points (14, 16, 19, and 22 years) with the Alcohol Use Disorder Identification Test (AUDIT; self-report version) and modeled using latent growth curve models (LGCM). Exposure variables were preselected based on publications^4,5,13,14,15,16,17,18,19,20^ regarding risk and resilience factors associated with alcohol misuse in adolescents and young adults. We aimed to cover a broad variety of domains, namely personality, social, and brain functioning characteristics, and familial risk for substance misuse. All assessments are listed in Table 1 and described in the eMethods in Supplement 1.

**Table 1.** Factors Associated With Alcohol Misuse According to Domains Assessed at Baseline and Respective Instruments

Domain	Measures	Features
Personality	Temperament and Character Inventory-Revised (TCI-R; novelty seeking subscale)	Impulsivity
	Neuroticism-Extraversion-Openness Personality Inventory-Revised (NEO-PI-R)	Extraversion
	Cambridge Guessing Task (CGT; from the Cambridge Neuropsychological Test Automated Battery [CANTAB])	Risk taking
Social factors	Parental socioeconomic status	
	Life Events Questionnaire (LEQ)	Frequencies of life events in family and sexuality domains
Brain functioning	Monetary incentive delay task (MID)	Reward outcome, reward anticipation, ventral striatum, and ventromedial prefrontal cortex
Family history	Familial risk for substance misuse	Family history of alcohol misuse and/or drug misuse

### Statistical Analyses

We used LGCM to analyze longitudinal within-participant as well as between-participant changes in alcohol misuse. LGCM analyses were conducted using Ωnyx (von Oertzen et al^21^) and descriptive analyses were conducted using SPSS version 28 (IBM).

**Figure 1.**
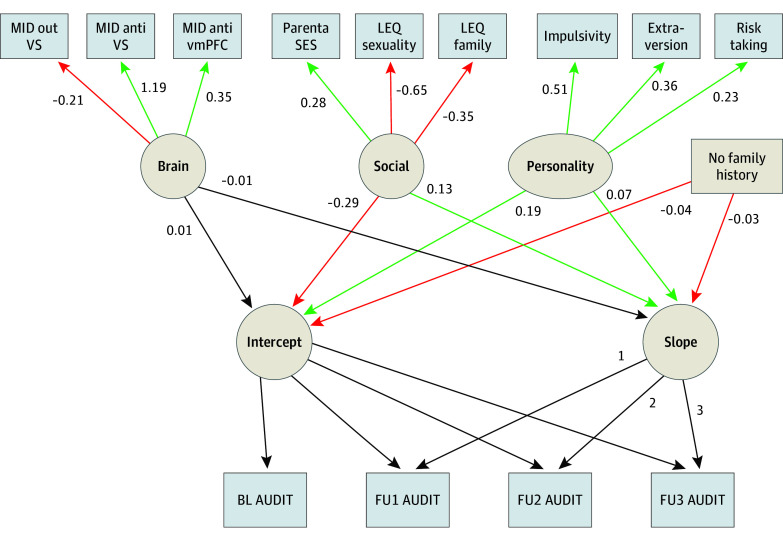
Latent Growth Curve Model Green paths show statistically significant positive associations and red paths show statistically significant negative associations. AUDIT indicates Alcohol Use Disorder Identification Test; BL, baseline; FU, follow-up; LEQ, Live Events Questionnaire; MID, monetary incentive delay; SES, socioeconomic status; vmPFC, ventromedial prefrontal cortex; VS, ventral striatum.

The LGCM (Figure 1) contained individual changes in AUDIT scores (within-person variability) and exposure variables associated with alcohol misuse (between-person variability). The modeled intercepts and slopes represent the individual 8-year trajectory of alcohol misuse, and the exposure variables allow for analysis of interindividual associations of these variables with misuse trajectories.

To address domain-specific dependency of the exposure variables, the brain, social, and personality domains were treated as latent factors in the model. Estimate parameters of the 4 exposure variables (ie, brain, social, personality, and family history) were statistically compared analogously to Clogg and colleagues^22^ and corrected for multiple testing.

Freely estimated covariances were allowed between all exposure variables (3 latent factors and family history). Maximum likelihood was used to estimate parameters, and a linear trajectory of AUDIT scores was assumed, which was verified by comparing the model fit to an analogous quadratic model and a model without time trend using Akaike information criterion (AIC). Models with higher AIC scores were considered to fit the time trend of observed AUDIT scores better.^23^ Missing data was handled with full information maximum likelihood. Small effect sizes were defined as an *r* value greater than 0.10 and less than 0.30.^24^ A 2-sided *P* < .05 was considered statistically significant. For the multiple comparisons of the etsimate parameters, the Bonferroni correction was used to adjust the significance level to *P* < .004. Data analysis was conducted from July 2021 to September 2022.

## Results

Sample characteristics including descriptive statistics on all analyzed variables are displayed in Table 2. The sample comprised 2240 participants (1110 female [49.6%] and 1130 male [50.4%]). Alcohol misuse (AUDIT) was measured in 2209 participants (98.6%) at study baseline (14 years), in 1698 (75.8%) participants at follow-up 1 (16 years; 511 participants [23.1%] dropped out from baseline), in 1511 participants (67.5%) at follow-up 2 (19 years; 729 participants [31.6%] dropped out from baseline) and in 1359 participants (60.7%) at follow-up 3 (22 years; 881 participants [38.5%] dropped out from baseline).

**Table 2.** Sample Characteristics

Characteristics	Observations, No. (%) (N = 2240)	Score, mean (SD)
Sex		
Female	1110 (49.6)	NA
Male	1130 (50.4)	NA
Family history (cluster with familial risk), No./total No. (%)	1187/2235 (53.0)	NA
Baseline assessments (14 y)		
MID: Outcome phase (ventral striatal activation)	1892 (84.5)	−0.24 (1.12)
MID: Anticipation phase (ventral striatal activation)	1942 (86.7)	0.22 (0.34)
MID: Anticipation phase (ventromedial prefrontal cortex activation)	1942 (86.7)	0.03 (0.62)
Parental socioeconomic status	2240 (100.0)	17.74 (3.86)
LEQ: sexuality domain (frequency score)	2240 (100.0)	0.29 (0.18)
LEQ: family domain (frequency score)	2240 (100.0)	0.26 (0.22)
Impulsivity	2206 (98.5)	26.79 (4.86)
Extraversion	2240 (100.0)	2.50 (0.46)
Risk taking	2240 (100.0)	0.55 (0.13)
Alcohol misuse (AUDIT)	2209 (98.6)	1.50 (2.67)
Follow-up		
1: AUDIT, 16 y	1698 (75.8)	3.72 (3.48)
2: AUDIT, 19 y	1511 (67.5)	5.64 (4.19)
3: AUDIT, 22 y	1359 (60.7)	6.12 (4.64)

Abbreviations: AUDIT, Alcohol Use Disorder Identification Test; LEQ, live events questionnaire; MID, monetary incentive delay; NA, not applicable.

**Figure 2.**
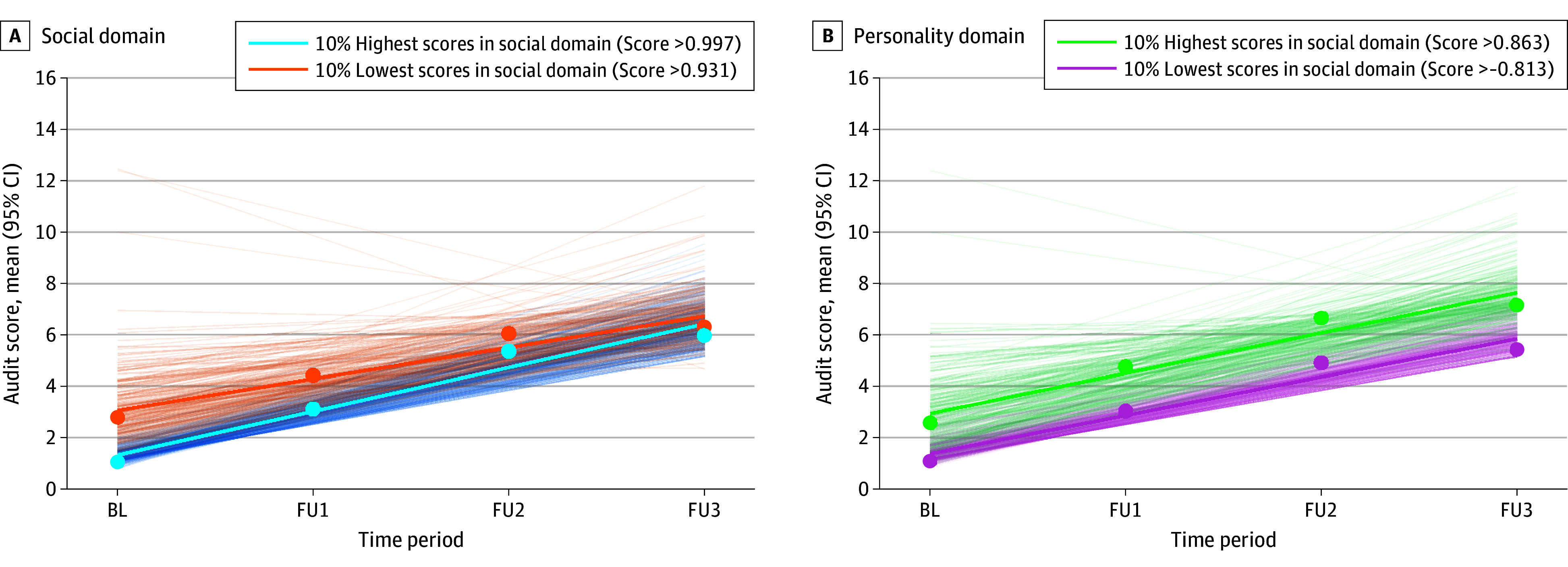
Alcohol Use Disorder Identification Test (AUDIT) Trajectories Visualized as Linear Regression Lines Based on AUDIT Raw Scores Grouped for Highest and Lowest Domain Scores A, The 10% highest and lowest scores in the social domain (socioeconomic status [SES] and life events); higher scores correspond to higher SES and lower frequency of life events related to family and sexuality. B, The 10% highest and lowest scores in the personality domain (impulsivity, extraversion, and risk-taking); higher scores correspond to higher impulsivity, extraversion, and risk-taking. Points indicate AUDIT group mean scores for each time point. Bold lines indicate mean regression lines based on AUDIT raw scores. Thin lines indicate individual regression lines based on AUDIT raw scores. BL indicates baseline; FU, follow-up.

The linear model showed an acceptable and better^23^ model fit in comparison with a quadratic model (−2 log likelihood ratio = 15.85; AIClinear = 77 694.85; AICquadratic = 77 710.70 and a better model fit in comparison with a model with no time trend (−2 log likelihood ratio = 222.73; AIClinear = 77 694.85; AICno-time-trend = 77 917.57) (Table 3). Regarding the factor paths (Table 3 and the eTable in Supplement 1), we found a significant negative association of the social domain with the intercept of alcohol misuse (β = −0.29; SE = 0.03; *P* < .001) and a significant positive association of the personality domain with the intercept (β = 0.19; SE = 0.03; *P* < .001), both of which had small effect sizes. Furthermore, there were significant positive associations of the social domain (β = 0.13; SE = 0.02; *P* < .001) and personality domains (β = 0.07; SE = 0.02; *P* < .001) with the slope of developmental trajectories of alcohol misuse, although these were small effect sizes. Family history also showed significant negative associations with the alcohol misuse intercept (β = −0.04; SE = 0.02; *P* = .045) and slope (β = −0.03; SE = 0.01; *P* = .01), but corresponding *r* coefficients suggested correlations beneath the threshold for a small effect size (Table 3). The latent factor, brain functioning, showed no association with intercept or slope of alcohol misuse. The 4 intercept estimates as well as the 4 slope estimates differed significantly (also after Bonferroni correction for multiple testing). Thus, social domain scores showed significantly larger-magnitude associations with the intercept and slope of alcohol misuse trajectories compared with the personality domain scores, which showed significantly larger-magnitude associations compared with the brain domain scores and family history of substance abuse. Figure 2A illustrates that higher social scores (higher socioeconomic status and lower frequency of life events related to family and sexuality) were associated with higher scores of alcohol misuse at baseline but were also associated with a greater slope over 8 years. Figure 2B shows that higher risk-taking aspects of personality were associated with higher scores of alcohol misuse at baseline, as well as a greater increase (slope) over 8 years.

**Table 3.** Intercept and Slope Model Estimates and Effect Sizes for the Brain, Social, Personality, and Family History Domains and Their Association With Alcohol Misuse

Domain	Intercept of alcohol misuse^a^				Slope of alcohol misuse^a^			
	β (SE)	*Z*	*P* value	*r*	β (SE)	*Z*	*P* value	*r*
Brain	0.01 (0.02)	0.54	.59	0.06	−0.01 (0.01)	−0.91	.36	−0.01
Social	−0.29 (0.03)	−8.72	<.001	−0.29^b^	0.13 (0.02)	7.39	<.001	0.18^b^
Personality	0.19 (0.04)	4.90	<.001	0.24^b^	0.07 (0.02)	3.61	<.001	0.12^b^
Family history	−0.04 (0.02)	−2.00	.045	−0.04	−0.03 (0.01)	−2.50	.01	−0.03

^a^ Latent growth curve model parameters: model fit: χ^2^_65_ = 258.74; *P* < .001; comparative fit index = 0.92; root mean square error of approximation = 0.04; standardized root mean squared residual = 0.03.

^b^ Values greater than 0.10 and less than 0.30 indicate a small effect size.

## Discussion

In this cohort study, we aimed to investigate the differential association of various domains with alcohol misuse trajectories from adolescence to young adulthood. The results of our final model indicate that psychosocial resources—indexed by lower frequency of life events related to family and sexuality, together with higher socioeconomic status—were initially associated with a lower general risk for alcohol misuse (intercept) but with increased alcohol misuse over the course of 8 years (slope). At the same time, personality characteristics—specifically higher impulsivity, risk-taking and extraversion scores—were associated with a higher risk of alcohol misuse on average, as well as a higher risk for alcohol misuse development over 8 years (slope). This finding suggests an influence of personality features on the general risk regarding alcohol misuse and on the development of alcohol misuse from adolescence to young adulthood. The absence of family history was significantly associated with a lower general risk and a decrease in alcohol misuse over time, with estimates below the threshold for a small effect size, which cannot be interpreted as meaningful. In earlier analyses of IMAGEN data,^10,11^ we saw a differential association of reward-related brain functioning at baseline with alcohol misuse 2 years later depending on family history of substance abuse.^13^ This finding suggests that family history of substance abuse and reward-system functioning may influence the course of adolescent alcohol misuse, specifically in certain combinations. Therefore, a possible mediating role of familial risk of the influence of reward processing on the course of alcohol misuse should be further investigated. Overall, the present analysis suggests a comparably large general association of social and personality factors with adolescent alcohol misuse that outweighs reward-system functioning and the individual familial risk. Thus, the risk of developing an alcohol addiction might primarily be influenced by social factors that in large parts are associated with the adolescent’s family as well as personality factors. Also, it has been shown that social and language functional magnetic resonance imaging tasks discriminate well on a neural level, suggesting that a broader range of brain processes might be associated with binge drinking in late adolescence.^8^

### Strengths and Limitations

The strength of the present study lies in our use of rich longitudinal data collected by the IMAGEN project to track the differential association of a diverse range of established factors with alcohol abuse over a broad neurodevelopmental window in a large sample. There were limitations to the study as well. First, limitations concern a selective bias due to dropouts that might have contributed to an underestimation of alcohol misuse.^25^ Second, alcohol misuse was only obtained via self-report; no external validation was administered. Third, as of yet, we have no information about whether participants with risky drinking trajectories will develop AUD in later life. Fourth, statistically the model showed an acceptable, but not a good fit, which is still highly informative for the field and future analyses.

## Conclusions

This cohort study found an association of social and personality factors with adolescent alcohol misuse compared with reward-related brain functioning and family history. This finding suggests that known risk factors for adolescent drinking may contribute differently to future alcohol misuse. This approach may inform more individualized preventive interventions.

## Notes

Supplement 1. **eMethods.** Description of Assessments **eTable.** Paths Estimates of Predictor Domains (Social, Personality, Brain)
eReferences

Supplement 2.
Nonauthor Collaborators

Supplement 3.
Data Sharing Statement
