# Supplementary material for: Personality, Social Factors, Brain Functioning, Familial Risk, and Trajectories of Alcohol Misuse in Adolescence
Source: JAMA Netw Open. 2024 Aug 16;7(8):e2425114. doi: 10.1001/jamanetworkopen.2024.25114 (PMC11329879; doi:10.1001/jamanetworkopen.2024.25114)
Supplement: Supplement 1. — eMethods. Description of Assessments eTable. Paths Estimates of Predictor Domains (Social, Personality, Brain) eReferences [file jamanetwopen-e2425114-s001.pdf]

Supplemental Online Content

Tschorn M, Daedelow L, Szalek L, et al; for the IMAGEN Consortium. Personality, social factors, brain functioning, and familial risk—trajectories of alcohol misuse in adolescence. *JAMA Netw Open*. 2024;7(8):e2425114. doi:10.1001/jamanetworkopen.2024.25114

eMethods. Description of Assessments
eTable. Paths Estimates of Predictor Domains (Social, Personality, Brain)
eReferences.

This supplemental material has been provided by the authors to give readers additional information about their work.

© 2024 Tschorn M et al. *JAMA Network Open.*

eMethods. Description of Assessments

Full description of assessments including fMRI tasks and study protocol can be found in Schumann et al., 2010.[1] Additional information regarding ethics, recruitment and standardized instructions for all tasks can be found in the publicly available standard operating procedure (https://imagen-project.org/?page_id=525). Assessments were associated with one of the four domains: brain predictors, social predictors, personality predictors and family history for substance abuse.

Personality predictors

Impulsivity. Impulsivity was measured using the revised version of the Temperament and Character Inventory (TCI-R).[2] The novelty seeking scale of the TCI-R was included to assess lower order trait dimensions more specifically related to disinhibitory psychopathology.

Extraversion. Extraversion was assessed as one dimension of the 60-item Neuroticism-Extraversion-Openness Five-Factor Inventory (NEO-PI-R) returning scores for Extraversion, Agreeableness, Conscientiousness, Neuroticism, and Openness to experience. The NEO-PI-R is a valid method of assessing broad dimensions of personality[3] based on the Five-Factor Model of personality.[4] The Extraversion factor measures the preference for seeking and engaging in social interactions and has been linked to sensitivity to rewarding environmental cues.[5]

Risk Taking. Risk taking was measured via the Cambridge Guessing Task (CGT) as part of the computerized Cambridge Neuropsychological Test Automated Battery (CANTAB).[6] The Cambridge Guessing Task (CGT) was developed to assess decision-making and risk-taking behavior outside a learning context. In the task, a row of ten boxes is presented at the top of a screen, some of which are red and some of which are blue. The bottom of the screen shows two rectangles containing the words 'Red' and 'Blue'. During each trial, participants are prompted to guess whether a yellow token is hidden behind a blue or a green box. In the gambling stages, subjects are given a number of points at the start, which is displayed on the screen. They can select a proportion of these points, displayed in either rising or falling order in a second box on the screen, to gamble on their confidence in this judgment. A stake box on the screen displays the current amount of the bet. The subject must try to accumulate as many points as possible. For IMAGEN, a modified version of the CGT was used, in which the time between stakes is reduced from 5s to 2s to make the task shorter and more interesting for adolescents. Stakes are displayed first in ascending, then in descending order.

Social predictors

Parental socioeconomic status (SES). Parental socioeconomic status score was derived from the parents' Development and Well-Being Assessment (DAWBA)[7] and European School Survey Project on Alcohol and Other Drugs questionnaires (ESPAD)[8] by adding the following variables: parental education, family stress, unemployment, financial difficulties, home inadequacy, neighborhood, financial crisis, parents' employment.[9] Negative scores were recoded so that higher scores would indicate a higher SES.

Life Events Questionnaire (LEQ). Stressful life events were assessed with the LEQ, a 39-item questionnaire asking about the lifetime occurrence and the perceived

© 2024 Tschorn M et al. *JAMA Network Open.*

valence of stressful events in the following domains: family/parents, accident/illness, sexuality, autonomy, deviance, relocation, and distress.[10] We used the two domains that are relevant for vulnerability and prediction of substance abuse: sexuality and family/parents. For analyses, the frequency scores of both domains were used.

Brain predictors

Monetary Incentive Delay (MID) Task. Brain predictors were extracted from fMRI data assessed during the Monetary Incentive Delay (MID) task. The MID is adapted from a previously described task[11] and requires participants to respond to a briefly presented target (250–400 ms) by pressing either a left-hand or right-hand button as quickly as possible to indicate whether the target appeared on the left or the right side of the screen. The target is presented after a delay of 4–4.5 s (blank screen) following a cue (250 ms). Participants scored points when responding while the target was on the screen, whereas they did not receive points for premature or delayed responses. A second cue before the onset of each trial reliably indicated the target position and the expected win in case of a successful response. A triangle as cue indicated no points, a one-lined circle 2 points and a three-lined circle 10 points. Twenty-two trials of each category (no win = 0 points, small win = 2 points, large win = 10 points) were presented in a pseudo-random order. The duration of the target was adjusted adaptively so that 66% of the trials produced a correct response. The participants were informed that they would receive one candy (M&M) for every five points won at the end of the session.

For functional fMRI analyses, contrast images for the anticipation period of large win minus no win, and the outcome period for large win minus no win were calculated. For the first-level analysis, experimental events were modeled by convolving the canonical hemodynamic response function with the onsets of the anticipation and feedback (win or fail) periods for each cue and feedback type as well as button presses. Individual contrast images were calculated for anticipation (large win versus small win) and outcome phase (large win versus no win) in hit trials. On the second level, these differential *t*-contrast images were entered to one-sample *t*-tests including scanning site as covariate. Regions of interest (ROI) analyses were conducted using literature-based ROIs of the functional key nodes ventral striatum (VS) and ventromedial prefrontal cortex (VMPFC). Brain activation in the VS during outcome and anticipation phase of the MID and brain activation in the vmPFC during anticipation phase of the MID were used for the latent growth curve modeling[9].

Familial risk

Familial risk for substance misuse. Familial risk of drug and alcohol misuse was a composite of multiple measurements and categorized in “positive family history” (score 2), “negative family history” (score 0), and “intermediate family history” (score 1, neither positive nor negative).[12] To assess familial risk of illicit drug and alcohol misuse, the following measurements were used: the Michigan Alcohol Screening Test (MAST), a family history interview on substance misuse, parent-administered AUDIT and the Drug Abuse Screening Test (DAST).[1] The criteria for intermediate family history of alcohol misuse or illicit drug use were met, if parents showed elevated scores on MAST, DAST, or AUDIT without clear indication for misuse or when alcohol or illicit drug misuse was assessed for second degree relatives. An intermediate family history of illicit drug misuse was identified when parents scored higher on DAST or drug misuse was assessed for second degree relatives or when family history of alcohol misuse was positive.

© 2024 Tschorn M et al. *JAMA Network Open.*

A positive family history of alcohol abuse was specified when at least one first degree relative was reported to suffer from alcohol abuse (family interview) or when parents' MAST score indicated an abuse of alcohol. A positive family history of illicit drug abuse was specified when at least one first degree relative was reported to suffer from drug abuse (family interview).

To group adolescents regarding their family history of substance abuse, a Two-Step Cluster analysis (TSC) was conducted using family history of substance misuse (alcohol and illicit drugs) as input variables. Resulting clusters regarding the family history variables were compared by calculating Mann–Whitney U tests. This data reduction approach was implemented in order to separate participants according to familial risk, assessed by two variables á three categories.

eTable. Paths Estimates of Predictor Domains (Social, Personality, Brain)

**Indicator**	**Domain**	**Estimate**	**SE**
MID outcome ventral striatum	Brain	-0.21	0.04
MID anticipation ventral striatum	Brain	1.19	0.18
MID anticipation ventromedial prefrontal cortex	Brain	0.35	0.06
Parental SES	Social	0.28	0.04
LEQ sexuality	Social	-0.65	0.05
LEQ family	Social	-0.35	0.04
Impulsivity (TCI-R)	Personality	0.51	0.04
Extraversion (NEO-PI-R)	Personality	0.36	0.03
Risk-taking (CGT)	Personality	0.23	0.03

Note: MID = Monetary Incentive Delay, VS = Ventral Striatum, SES = socioeconomic status, LEQ = Live Events Questionnaire, TCI-R = revised version of the Temperament and Character Inventory, NEO-PI-R = Neuroticism-Extraversion-Openness Five-Factor Inventory, CGT = Cambridge Guessing Task

© 2024 Tschorn M et al. *JAMA Network Open.*

eReferences

1. Schumann, G., Loth, E., Banaschewski, T., Barbot, A., Barker, G., Büchel, C., Conrod, P., Dalley, J., Flor, H., & Gallinat, J. (2010). The IMAGEN study: Reinforcement-related behaviour in normal brain function and psychopathology. *Molecular Psychiatry*, *15*(12), 1128–1139.
2. Cloninger, C. R. (1999). The temperament and character inventory-revised. *St Louis, MO: Center for Psychobiology of Personality, Washington University.*
3. De Fruyt, F., Van de Wiele, L., & Van Heeringen, C. (2000). Cloninger's psychobiological model of temperament and character and the five-factor model of personality. *Personality and Individual Differences*, *29*(3), 441–452.
4. Costa Jr, P. T., & McCrae, R. R. (1995). Domains and facets: Hierarchical personality assessment using the Revised NEO Personality Inventory. *Journal of Personality Assessment*, *64*(1), 21–50.
5. Watson, D., & Clark, L. A. (1992). On traits and temperament: General and specific factors of emotional experience and their relation to the five-factor model. *Journal of Personality*, *60*(2), 441–476.
6. Cambridge Cognition. (2019). *CANTAB®, Cognitive assessment software*. Cambridge Cognition. www.cantab.com
7. Goodman, R., Ford, T., Richards, H., Gatward, R., & Meltzer, H. (2000). The development and well-being assessment: Description and initial validation of an integrated assessment of child and adolescent psychopathology. *Journal of Child Psychology and Psychiatry*, *41*(5), 645–655.
8. Hibell, B., Andersson, B., Bjarnason, T., Kokkevi, A., Morgan, M., Narusk, A., & Ahlström, S. (1997). *The 1995 ESPAD report. Alcohol and other drug use among students in 26 European countries.*
9. Tschorn, M., Lorenz, R. C., O'Reilly, P. F., Reichenberg, A., Banaschewski, T., Bokde, A. L., Quinlan, E. B., Desrivières, S., Flor, H., & Grigis, A. (2021). Differential

© 2024 Tschorn M et al. *JAMA Network Open.*

predictors for alcohol use in adolescents as a function of familial risk. *Translational Psychiatry*, *11*(1), 1–11.

10. Newcomb, M. D., Huba, G. J., & Bentler, P. M. (1981). A multidimensional assessment of stressful life events among adolescents: Derivation and correlates. *Journal of Health and Social Behavior*, 400–415.
11. Knutson, B., Fong, G. W., Adams, C. M., Varner, J. L., & Hommer, D. (2001). Dissociation of reward anticipation and outcome with event-related fMRI. *Neuroreport*, *12*(17), 3683–3687.
12. Whelan, R., Watts, R., Orr, C. A., Althoff, R. R., Artiges, E., Banaschewski, T., Barker, G. J., Bokde, A. L., Büchel, C., & Carvalho, F. M. (2014). Neuropsychosocial profiles of current and future adolescent alcohol misusers. *Nature*, *512*(7513), 185–189.

© 2024 Tschorn M et al. *JAMA Network Open.*
