# Supplementary material for: Personality, Social Factors, Brain Functioning, Familial Risk, and Trajectories of Alcohol Misuse in Adolescence
Source: JAMA Netw Open. 2024 Aug 16;7(8):e2425114. doi: 10.1001/jamanetworkopen.2024.25114 (PMC11329879; doi:10.1001/jamanetworkopen.2024.25114)
Supplement: Supplement 2. — Nonauthor Collaborators [file jamanetwopen-e2425114-s002.pdf]

Supplemental Online Content: Nonauthor Collaborators
*First name, last name, and suffix (if applicable) are required and will appear in PubMed.

## *Group Name(s): IMAGEN consortium

| ***First Name and Middle Initial(s)** | ***Last Name** | ***Suffix (eg, Jr, III)** | Academic Degrees | Institution | Location (city, state/province, country) | Role or Contribution, eg, chair, principal investigator | Group (if more than 1 Group listed in the byline) and/or Subgroup (eg, Steering Committee) |
|---|---|---|---|---|---|---|---|
| Trevor | Robbin | | PhD | Behavioural and Clinical Neuroscience Institute | Cambridge University, Cambridge, UK | acquisition, analysis, or interpretation of data | |
| Jeffrey | Dalley | | PhD | Behavioural and Clinical Neuroscience Institute | Cambridge University, Cambridge, UK | acquisition, analysis, or interpretation of data | |
| Naresh | Subramaniam | | PhD | Behavioural and Clinical Neuroscience Institute | Cambridge University, Cambridge, UK | acquisition, analysis, or interpretation of data | |
| David | Theobald | | MSc | Behavioural and Clinical Neuroscience Institute | Cambridge University, Cambridge, UK | acquisition, analysis, or interpretation of data | |
| Karl | Mann | | PhD | Central Institute of Mental Healt | Mannheim, Germany | acquisition, analysis, or interpretation of data | |
| Christiane | Bach | | MSc | Department of Child and Adolescent Psychiatry andPsy | Mannheim, Germany | acquisition, analysis, or interpretation of data | |
| Maren | Struve | | PhD | Central Institute of Mental Health | Mannheim, Germany | acquisition, analysis, or interpretation of data | |
| Marcella | Rietschel | | PhD | Central Institute of Mental Health | Mannheim, Germany | acquisition, analysis, or interpretation of data | |
| Rainer | Spanagel | | PhD | Central Institute of Mental Health | Mannheim, Germany | acquisition, analysis, or interpretation of data | |
| Mira | Fauth-Bühler | | PhD | Central Institute of Mental Health | Mannheim, Germany | acquisition, analysis, or interpretation of data | |
| Sabina | Millenet | | PhD | Department of Child and Adolescent Psychiatry andPsy | Mannheim, Germany | acquisition, analysis, or interpretation of data | |
| Yvonne | Grimmer | | PhD | Central Institute of Mental Health | Mannheim, Germany | acquisition, analysis, or interpretation of data | |
| Mark | Lathrop | | PhD | Centre National de Génotypage, Genoscope | Èvry, France | acquisition, analysis, or interpretation of data | |
| Lisa | Albrecht | | MSc | Department ofPsychiatry and Psychotherapy CCM, Ch | Berlin, Germany | acquisition, analysis, or interpretation of data | |
| Nikolay | Ivanov | | | Department ofPsychiatry and Psychotherapy CCM, Ch | Berlin, Germany | acquisition, analysis, or interpretation of data | |
| Nicole | Strache | | PhD | Department ofPsychiatry and Psychotherapy CCM, Ch | Berlin, Germany | acquisition, analysis, or interpretation of data | |
| Andreas | Ströhle | | PhD | Department ofPsychiatry and Psychotherapy CCM, Ch | Berlin, Germany | acquisition, analysis, or interpretation of data | |
| Jan | Reuter | | PhD | Department ofPsychiatry and Psychotherapy CCM, Ch | Berlin, Germany | acquisition, analysis, or interpretation of data | |
| Jürgen | Gallinat | | PhD | Department ofPsychiatry and Psychotherapy CCM, Ch | Berlin, Germany | acquisition, analysis, or interpretation of data | |
| Isabel | Gemmeke | | | Department ofPsychiatry and Psychotherapy CCM, Ch | Berlin, Germany | acquisition, analysis, or interpretation of data | |
| Alexander | Genauck | | PhD | Department ofPsychiatry and Psychotherapy CCM, Ch | Berlin, Germany | acquisition, analysis, or interpretation of data | |
| Caroline | Parchetka | | PhD | Department ofPsychiatry and Psychotherapy CCM, Ch | Berlin, Germany | acquisition, analysis, or interpretation of data | |
| Katharina | Weiß | | | Department ofPsychiatry and Psychotherapy CCM, Ch | Berlin, Germany | acquisition, analysis, or interpretation of data | |
| Johann | Kruschwitz | | PhD | Department ofPsychiatry and Psychotherapy CCM, Ch | Berlin, Germany | acquisition, analysis, or interpretation of data | |
| Bianca | Raffaelli | | MD | Department ofPsychiatry and Psychotherapy CCM, Ch | Berlin, Germany | acquisition, analysis, or interpretation of data | |
| Alexis | Barbot | | PhD | Comissariat à l'Energie Atomique | Fontenay-aux-Roses, Franc | acquisition, analysis, or interpretation of data | |
| Benjamin | Thyreau | | | Comissariat à l'Energie Atomique | Fontenay-aux-Roses, Franc | acquisition, analysis, or interpretation of data | |
| Yannick | Schwartz | | | Comissariat à l'Energie Atomique | Fontenay-aux-Roses, Franc | acquisition, analysis, or interpretation of data | |
| Christophe | Lalanne | | | Comissariat à l'Energie Atomique | Fontenay-aux-Roses, Franc | acquisition, analysis, or interpretation of data | |

*First name, last name, and suffix (if applicable) are required and will appear in PubMed.

| *First Name and Middle Initial(s) | *Last Name | *Suffix (eg, Jr, III) | Academic Degrees | Institution | Location (city, state/province, country) | Role or Contribution, eg, chair, principal investigator | Group (if more than 1 Group listed in the byline) and/or Subgroup (eg, Steering Committee) |
|---|---|---|---|---|---|---|---|
| Vincent Frouin25, | Frouin | | PhD | Comissariat à l'Energie Atomique | Fontenay-aux-Roses, Franc | acquisition, analysis, or interpretation of data | |
| John | Rogers | | | Delosis | Twickenham, UK | acquisition, analysis, or interpretation of data | |
| James | Ireland | | | Delosis | Twickenham, UK | acquisition, analysis, or interpretation of data | |
| Dirk | Lanzerath | | PhD | Deutsches Referenzzentrum für Ethik | Bonn, Germany | acquisition, analysis, or interpretation of data | |
| Jianfeng | Feng | | PhD | Fudan University | Shanghai, China | acquisition, analysis, or interpretation of data | |
| Zuleima | Bricaud | | | InstitutNational de la Santé et de la Recherche Médical | Gif-sur-Yvette, France | acquisition, analysis, or interpretation of data | |
| Fanny | Gollier Briand | | | InstitutNational de la Santé et de la Recherche Médical | Gif-sur-Yvette, France | acquisition, analysis, or interpretation of data | |
| Hervé | LemaÎtre | | PhD | Group d'Imagerie Neurofonctionelle, Institut de Malad | Bordeaux, France | acquisition, analysis, or interpretation of data | |
| Ruben | Miranda | | | InstitutNational de la Santé et de la Recherche Médical | Gif-sur-Yvette, France | acquisition, analysis, or interpretation of data | |
| Jessica | Massicotte | | | InstitutNational de la Santé et de la Recherche Médical | Gif-sur-Yvette, France | acquisition, analysis, or interpretation of data | |
| Helene | Vulser | | | InstitutNational de la Santé et de la Recherche Médical | Gif-sur-Yvette, France | acquisition, analysis, or interpretation of data | |
| Jani | Pentillä | | PhD | InstitutNational de la Santé et de la Recherche Médical | Gif-sur-Yvette, France | acquisition, analysis, or interpretation of data | |
| Irina | Filippi | | | InstitutNational de la Santé et de la Recherche Médical | Gif-sur-Yvette, France | acquisition, analysis, or interpretation of data | |
| André | Galinowski | | | InstitutNational de la Santé et de la Recherche Médical | Gif-sur-Yvette, France | acquisition, analysis, or interpretation of data | |
| Pauline | Bezivin | | | InstitutNational de la Santé et de la Recherche Médical | Gif-sur-Yvette, France | acquisition, analysis, or interpretation of data | |
| Anna | Cattrell | | PhD | Social, Genetic andDevelopmental Psychiatry Centre, I | London, UK | acquisition, analysis, or interpretation of data | |
| Tianye | Jia | | PhD | Social, Genetic andDevelopmental Psychiatry Centre, I | London, UK | acquisition, analysis, or interpretation of data | |
| Helen | Werts | | | Social, Genetic andDevelopmental Psychiatry Centre, I | London, UK | acquisition, analysis, or interpretation of data | |
| Lauren | Topper | | | Social, Genetic andDevelopmental Psychiatry Centre, I | London, UK | acquisition, analysis, or interpretation of data | |
| Laurence | Reed | | PhD | Social, Genetic andDevelopmental Psychiatry Centre, I | London, UK | acquisition, analysis, or interpretation of data | |
| Chris | Andrew | | | Social, Genetic andDevelopmental Psychiatry Centre, I | London, UK | acquisition, analysis, or interpretation of data | |
| Catherine | Mallik | | | Social, Genetic andDevelopmental Psychiatry Centre, I | London, UK | acquisition, analysis, or interpretation of data | |
| Barbara | Ruggeri | | PhD | Social, Genetic andDevelopmental Psychiatry Centre, I | London, UK | acquisition, analysis, or interpretation of data | |
| Gareth | Barker | | PhD | Social, Genetic andDevelopmental Psychiatry Centre, I | London, UK | acquisition, analysis, or interpretation of data | |
| Charlotte | Nymberg | | PhD | Social, Genetic andDevelopmental Psychiatry Centre, I | London, UK | acquisition, analysis, or interpretation of data | |
| Patricia J. | Conrod | | PhD | Social, Genetic andDevelopmental Psychiatry Centre, I | London, UK | acquisition, analysis, or interpretation of data | |
| Lindsay | Smith | | PhD | Social, Genetic andDevelopmental Psychiatry Centre, I | London, UK | acquisition, analysis, or interpretation of data | |
| Eva | Loth | | PhD | Social, Genetic andDevelopmental Psychiatry Centre, I | London, UK | acquisition, analysis, or interpretation of data | |
| Stephanie | Havatzias | | | Social, Genetic andDevelopmental Psychiatry Centre, I | London, UK | acquisition, analysis, or interpretation of data | |
| Emily | Kitson | | | Social, Genetic andDevelopmental Psychiatry Centre, I | London, UK | acquisition, analysis, or interpretation of data | |
| Alice | Robinson | | | Social, Genetic andDevelopmental Psychiatry Centre, I | London, UK | acquisition, analysis, or interpretation of data | |

Supplemental Online Content: Nonauthor Collaborators

*First name, last name, and suffix (if applicable) are required and will appear in PubMed.

| *First Name and Middle Initial(s) | *Last Name | *Suffix (eg, Jr, III) | Academic Degrees | Institution | Location (city, state/province, country) | Role or Contribution, eg, chair, principal investigator | Group (if more than 1 Group listed in the byline) and/or Subgroup (eg, Steering Committee) |
|---|---|---|---|---|---|---|---|
| Deborah | Hall | | | Social, Genetic andDevelopmental Psychiatry Centre, I | London, UK | acquisition, analysis, or interpretation of data | |
| Chiara | Rubino | | BSc | Social, Genetic andDevelopmental Psychiatry Centre, I | London, UK | acquisition, analysis, or interpretation of data | |
| Hannah | Wright | | PhD | Social, Genetic andDevelopmental Psychiatry Centre, I | London, UK | acquisition, analysis, or interpretation of data | |
| Kerstin | Stueber | | | Social, Genetic andDevelopmental Psychiatry Centre, I | London, UK | acquisition, analysis, or interpretation of data | |
| Eanna | Hanratty | | | Social, Genetic andDevelopmental Psychiatry Centre, I | London, UK | acquisition, analysis, or interpretation of data | |
| Eleanor | Kennedy | | | Social, Genetic andDevelopmental Psychiatry Centre, I | London, UK | acquisition, analysis, or interpretation of data | |
| Fabiana Mesquita | de Carvahlo | | PhD | Social, Genetic andDevelopmental Psychiatry Centre, I | London, UK | acquisition, analysis, or interpretation of data | |
| Argyris | Stringaris | | PhD, MD | Social, Genetic andDevelopmental Psychiatry Centre, I | London, UK | acquisition, analysis, or interpretation of data | |
| Alex | Ing | | PhD | Social, Genetic andDevelopmental Psychiatry Centre, I | London, UK | acquisition, analysis, or interpretation of data | |
| Gabriel | Robert | | PhD, MD | Social, Genetic andDevelopmental Psychiatry Centre, I | London, UK | acquisition, analysis, or interpretation of data | |
| Christine | Macare | | PhD | Social, Genetic andDevelopmental Psychiatry Centre, I | London, UK | acquisition, analysis, or interpretation of data | |
| Bing | Xu | | | Social, Genetic andDevelopmental Psychiatry Centre, I | London, UK | acquisition, analysis, or interpretation of data | |
| Tao | Yu | | PhD, MD | Social, Genetic andDevelopmental Psychiatry Centre, I | London, UK | acquisition, analysis, or interpretation of data | |
| Erin | Burke Quinlan | | PhD | Social, Genetic andDevelopmental Psychiatry Centre, I | London, UK | acquisition, analysis, or interpretation of data | |
| Patrick | Constant | | | Pertimm | Paris, France | acquisition, analysis, or interpretation of data | |
| Semiha | Aydin | | | Physikalisch-TechnischeBundesanstalt (PTB), Braunsc | Berlin, Germany | acquisition, analysis, or interpretation of data | |
| Ruediger | Brühl | | PhD | Physikalisch-TechnischeBundesanstalt (PTB), Braunsc | Berlin, Germany | acquisition, analysis, or interpretation of data | |
| Albrecht | Ihlenfeld | | | Physikalisch-TechnischeBundesanstalt (PTB), Braunsc | Berlin, Germany | acquisition, analysis, or interpretation of data | |
| Bernadeta | Walaszek | | | Physikalisch-TechnischeBundesanstalt (PTB), Braunsc | Berlin, Germany | acquisition, analysis, or interpretation of data | |
| Bernd | Ittermann | | PhD | Physikalisch-TechnischeBundesanstalt (PTB), Braunsc | Berlin, Germany | acquisition, analysis, or interpretation of data | |
| Michael | Smolka | | PhD | Department of Psychiatry and NeuroimagingCenter, Te | Dresden, Germany | acquisition, analysis, or interpretation of data | |
| Thomas | Hübner | | PhD | Department of Psychiatry and NeuroimagingCenter, Te | Dresden, Germany | acquisition, analysis, or interpretation of data | |
| Kathrin | Müller | | | Department of Psychiatry and NeuroimagingCenter, Te | Dresden, Germany | acquisition, analysis, or interpretation of data | |
| Stephan | Ripke | | PhD | Department of Psychiatry and NeuroimagingCenter, Te | Dresden, Germany | acquisition, analysis, or interpretation of data | |
| Sarah | Jurk | | | Department of Psychiatry and NeuroimagingCenter, Te | Dresden, Germany | acquisition, analysis, or interpretation of data | |
| Eva | Mennigen | | | Department of Psychiatry and NeuroimagingCenter, Te | Dresden, Germany | acquisition, analysis, or interpretation of data | |
| Dirk | Schmidt | | | Department of Psychiatry and NeuroimagingCenter, Te | Dresden, Germany | acquisition, analysis, or interpretation of data | |
| Nora | Vetter | | PhD | Department of Psychiatry and NeuroimagingCenter, Te | Dresden, Germany | acquisition, analysis, or interpretation of data | |
| Veronika | Ziesch | | | Department of Psychiatry and NeuroimagingCenter, Te | Dresden, Germany | acquisition, analysis, or interpretation of data | |
| Juliane H. | Fröhner | | MSc | Department of Psychiatry and NeuroimagingCenter, Te | Dresden, Germany | acquisition, analysis, or interpretation of data | |
| Daniel | Carter | | | Trinity College Dublin | Dublin, Ireland | acquisition, analysis, or interpretation of data | |

Supplemental Online Content: Nonauthor Collaborators
*First name, last name, and suffix (if applicable) are required and will appear in PubMed.

| *First Name and Middle Initial(s) | *Last Name | *Suffix (eg, Jr, III) | Academic Degrees | Institution | Location (city, state/province, country) | Role or Contribution, eg, chair, principal investigator | Group (if more than 1 Group listed in the byline) and/or Subgroup (eg, Steering Committee) |
|---|---|---|---|---|---|---|---|
| Susanne | O'Driscoll | | | Trinity College Dublin | Dublin, Ireland | acquisition, analysis, or interpretation of data | |
| Emily | Walsh | | | Trinity College Dublin | Dublin, Ireland | acquisition, analysis, or interpretation of data | |
| Maria Leonora Fatir | Agan | | MSc | Trinity College Dublin | Dublin, Ireland | acquisition, analysis, or interpretation of data | |
| Mairead | McMorrow | | | Trinity College Dublin | Dublin, Ireland | acquisition, analysis, or interpretation of data | |
| Sinead | Nugent | | PhD | Trinity College Dublin | Dublin, Ireland | acquisition, analysis, or interpretation of data | |
| Colm | Connolly | | | Trinity College Dublin | Dublin, Ireland | acquisition, analysis, or interpretation of data | |
| Eoin | Dooley | | MSc | Trinity College Dublin | Dublin, Ireland | acquisition, analysis, or interpretation of data | |
| Clodagh | Cremen | | PhD | Trinity College Dublin | Dublin, Ireland | acquisition, analysis, or interpretation of data | |
| Jennifer | Jones | | PhD | University College Dublin | Dublin, Ireland | acquisition, analysis, or interpretation of data | |
| John | O'Keefe | | BA | University College Dublin | Dublin, Ireland | acquisition, analysis, or interpretation of data | |
| Martin | O'Connor | | | University College Dublin | Dublin, Ireland | acquisition, analysis, or interpretation of data | |
| Jean-Baptiste | Poline | | PhD | 34 McGill University and University of California | Montréal, Canada and Berl | acquisition, analysis, or interpretation of data | |
| Uli | Bromberg | | PhD | University Medical Centre Hamburg-Eppendorf | Hamburg, Germany | acquisition, analysis, or interpretation of data | |
| Tahmine | Fadai | | MD | University Medical Centre Hamburg-Eppendorf | Hamburg, Germany | acquisition, analysis, or interpretation of data | |
| Juliana | Yacubian | | PhD | University Medical Centre Hamburg-Eppendorf | Hamburg, Germany | acquisition, analysis, or interpretation of data | |
| Sophia | Schneider | | PhD | University Medical Centre Hamburg-Eppendorf | Hamburg, Germany | acquisition, analysis, or interpretation of data | |
| Maria | Lobatcheva | | | University Medical Centre Hamburg-Eppendorf | Hamburg, Germany | acquisition, analysis, or interpretation of data | |
| Claire | Lawrence | | PhD | University of Nottingham, University Park, | Nottingham, UK | acquisition, analysis, or interpretation of data | |
| Craig | Newman | | | University of Nottingham, University Park, | Nottingham, UK | acquisition, analysis, or interpretation of data | |
| Kay | Head | | | University of Nottingham, University Park, | Nottingham, UK | acquisition, analysis, or interpretation of data | |
| Alicia | Stedman | | | University of Nottingham, University Park, | Nottingham, UK | acquisition, analysis, or interpretation of data | |
| Nadja | Heym | | PhD | University of Nottingham, University Park, | Nottingham, UK | acquisition, analysis, or interpretation of data | |
| Mehri | Kaviani | | PhD | University of Nottingham, University Park, | Nottingham, UK | acquisition, analysis, or interpretation of data | |
| Tomáš | Paus | | PhD | University of Toronto | Toronto, ON, Canada | acquisition, analysis, or interpretation of data | |
| Dai | Stephens | | PhD | University of Sussex | Brighton, UK | acquisition, analysis, or interpretation of data | |
| Zdenka | Pausova | | PhD | University of Toronto | Toronto, ON, Canada | acquisition, analysis, or interpretation of data | |
| Amir | Tahmasebi | | PhD | University of Toronto | Toronto, ON, Canada | acquisition, analysis, or interpretation of data | |
