# Supplementary material for: Personality, Social Factors, Brain Functioning, Familial Risk, and Trajectories of Alcohol Misuse in Adolescence
Source: JAMA Netw Open. 2024 Aug 16;7(8):e2425114. doi: 10.1001/jamanetworkopen.2024.25114 (PMC11329879; doi:10.1001/jamanetworkopen.2024.25114)
Supplement: Supplement 3. — Data Sharing Statement [file jamanetwopen-e2425114-s003.pdf]

Data Sharing Statement

Tschorn. Personality, Social Factors, Brain Functioning, and Familial Risk—Trajectories of Alcohol Misuse in Adolescence. *JAMA Netw Open*. Published August 16, 2024.
doi:10.1001/jamanetworkopen.2024.25114

Data
Data available: No
